# Supplementary material for: COVID-19 prevention is shaped by polysocial risk: A cross-sectional study of vaccination and testing disparities in underserved populations
Source: PLoS One. 2025 Jul 17;20(7):e0328779. doi: 10.1371/journal.pone.0328779 (PMC12270183; doi:10.1371/journal.pone.0328779)
Supplement: S1 Table — This table provides detailed metadata for various study projects, including geographic location, primary and secondary populations, study design, study setting, mode of data collection, and vaccine availability phase. It also includes exclusion criteria for the study population and descriptions of prevention behaviors and population characteristics. (DOCX) [file pone.0328779.s001.docx]

| **Supplemental Table 1**  Study Project Metadata | | | | | | | | |
| --- | --- | --- | --- | --- | --- | --- | --- | --- |
| **Project** | **Geographic**  **Location** | **Primary**  **Population** | **Secondary Population** | **Study**  **Design** | **Study**  **Setting** | **Mode of**  **Data Collection** | **Study Data Collection Start Date** | **Vaccine Availability Phase** |
| **P1** | Delaware | Low income populations, African American, Hispanic / LatinX | N/A | Prospective observational study case reports | Community centers, Other community (non-clinical) settings, Facilities convenient to participants homes | Interview, Questionnaire/survey (in-person), COVID-19 testing, Other biological data (e.g. blood sample, nasal swab) | 3/4/2021 | Phase 1 - First vaccine delivered and available to certain populations (between 12/14/20 and 04/19/21) |
| **P2** | Maryland | African American, Hispanic / LatinX | N/A | Randomized controlled trial | Home (e.g. mailed and email surveys, in-home interviews), Outpatient clinic, Mobile testing unit | Interview,  Questionnaire/survey (online or by mail) COVID-19 testing, Other biological data (e.g. blood sample, nasal swab) | 2/17/2021 | Phase 1 - First vaccine delivered and available to certain populations (between 12/14/20 and 04/19/21 |
| **P3** | New York | Low income housing | N/A | Randomized controlled trial, Cluster randomized trial | Home (e.g. mailed and email surveys, in-home interviews) | Interview, COVID-19 testing, Qualitative focus group | 8/2/2021 | Phase 2 - First vaccine available to all (between 04/20/21 and 09/25/21) |
| **P4** | Florida | African American,  Hispanic / LatinX, Older Adults, Substance Users, Low income populations, Low income housing,  People Experiencing Homelessness,  Federally Qualified Health Centers (FQHCs), People living with HIV / AIDS (PLWHA) | N/A | Cross sectional cohort | Community centers, Other community (non-clinical) settings - In collaboration with community partners we tested participants at schools, churches, community centers, Mobile testing unit, Community health center | Interview,  Questionnaire/survey (online or by mail), COVID-19 testing, Other biological data (e.g. blood sample, nasal swab), Electronic Health Records, COVID-19 tests data from Electronic Health ecords/Secondary Medical Records | 5/18/2021 | Phase 2 - First vaccine available to all (between 04/20/21 and 09/25/21) |
| **P5** | Illinois | Federally Qualified Health Centers (FQHCs) | Testing Deserts | Prospective observational cohort | Outpatient clinic, Hospital, Other community (non-clinical) settings | COVID-19 testing Questionnaire/survey (in-person), Questionnaire/survey (online or by mail) | 4/19/2021 | Phase 1 - First vaccine delivered and available to certain populations (between 12/14/20 and 04/19/21 |
| **P6** | New York | Substance users | Low income populations | Cross-sectional cohort Prospective Observational Cohort | Other community (non-clinical) settings (specify): areas served by two community-based organizations (CBOs) specializing in substance use and substance use disorders (SUD) treatment and providing support services for individuals who abuse substances | Interview, Questionnaire/survey (in-person), Questionnaire/survey (online or by mail),  Qualitative focus group, COVID-19 testing, Other biological data (nasal swab), Electronic Health Records | 6/7/2021 | Phase 2 - First vaccine available to all (between 04/20/21 and 09/25/21) |
| **P7** | North Carolina | Hispanic / LatinX, African American, Rural Populations | Farm workers | Case reports | Mobile testing units | Interview (Zoom), Qualitative focus group, Questionnaire | 10/21/2021 | Phase 3 - First booster available to certain populations (between 09/26/21 and 11/21/21) |
| **P8** | New York | Intellectual and Developmental Disabilities | Women experiencing homelessness | Prospective observational Cohort | Schools | Interview, Questionnaire/survey (in-person), Questionnaire/survey (online or by mail), COVID-19 testing | 5/11/2021 | Phase 2 - First vaccine available to all (between 04/20/21 and 09/25/21) |
| **P9** | Arizona | Hispanic / LatinX, Lower income population | N/A | Cross sectional cohort Cluster randomized trial | Schools | Questionnaire/survey (in-person), Questionnaire/survey (online or by mail),  Covid-19 testing | 11/30/2021 | Phase 4 - First booster available to all (between 11/22/21 and 05/23/22) |
| **P10** | California | Hispanic / LatinX, Low income populations | N/A | Prospective observational cohort study | Schools, Other home, COVID -19 testing | COVID-19 testing,  COVID-19 tests data from Electronic Health Records / Secondary medical records,  Questionnaire/survey (by phone) | 10/18/2021 | Phase 3 - First booster available to certain populations (between 09/26/21 and 11/21/21) |
| **P11** | Florida | African American, Hispanic / LatinX, Immigrants, Older Adults, LBGTQ/Sexual and Gender Minority (SGM), Substance Users, Low income populations, Low income housing, People Experiencing Homelessness, People living with HIV/AIDS (PLWHA) | N/A | Prospective observational cohort study Cross-sectional cohort | Community centers, Other community (non-clinical) settings, School, Other community (non-clinical) settings, Church, Outpatient clinic, Mobile testing unit, Community health center | Interview,  Questionnaire/survey (in-person), COVID-19 testing, Other biological data (e.g. blood sample, nasal swab), Electronic Health Records | 5/24/2022 | Post Phase 4 - Vaccine available to all (after 05/23/22) |
| **P12** | Oregon | Substance users | People Experiencing Homelessness  People living with HIV/AIDS (PLWHA) | Cross-sectional cohort | Community centers | Questionnaire/survey/(in-person), COVID-19 testing | 3/14/2022 | Phase 4 - First booster available to all (between 11/22/21 and 05/23/22) |
| **P13** | California | Substance users | N/A | Prospective observational cohort  Randomized controlled trial | Other community non-clinical  Mobile testing unit | Questionnaire/survey (in-person),  Interview, COVID-19 testing | 3/15/2022 | Phase 4 - First booster available to all (between 11/22/21 and 05/23/22) |
| **P14** | Arkansas | Hispanic / LatinX | N/A | Cross sectional cohort | Mobile testing unit, Community centers, Other community setting (community events-cultural events, holiday celebrations etc.) | Online, Questionnaire/survey, COVID-19 testing | 3/18/2022 | Phase 4 - First booster available to all (between 11/22/21 and 05/23/22) |
