# Supplementary material for: COVID-19 prevention is shaped by polysocial risk: A cross-sectional study of vaccination and testing disparities in underserved populations
Source: PLoS One. 2025 Jul 17;20(7):e0328779. doi: 10.1371/journal.pone.0328779 (PMC12270183; doi:10.1371/journal.pone.0328779)
Supplement: S2 Table — This table describes prevention behaviors and population characteristics, including survey questions and derivation details for primary outcomes such as COVID-19 vaccination, testing, and positive test results. It also covers demographics, economic risk characteristics, health risk characteristics, and access to COVID-19 testing. (DOCX) [file pone.0328779.s002.docx]

| **Supplemental Table 2**  Description of Prevention Behaviors and Population Characteristics | |
| --- | --- |
| **Characteristic** | **Survey Question Text & Derivation Details** |
| Primary Outcomes |  |
| Received COVID-19 Vaccination | Have you received a COVID-19 vaccine? |
| Tested for COVID-19 | Have you ever been tested for COVID-19? |
| Tested Positive for COVID-19 | Have you ever tested positive for COVID-19? This question is only applicable to participants who have previously been tested for COVID-19. |
|  |  |
| Demographics |  |
| Age | What is your age in years? |
| Sex | What was your sex assigned at birth on your birth certificate? |
| Race/Ethnicity | Derived as a composite of the following two questions:   1. What is your race? 2. Are you of Hispanic, Latino, or Spanish origin? |
| Geographic Region | Derived based on U.S. State or Territory. If no State data provided, then State is imputed from zip code, county, or project location. |
|  |  |
| Economic Risk Characteristics |  |
| Education | What is the highest level of education you have achieved outside or in the United States? Grades roughly equivalent to years of school. |
| Household Income | In 2019, what was your total household income before taxes?  Less than $15,000  $15,000 - $19,999  $20,000 - $24,999  $25,000 - $34,999  $35,000 - $49,999  $50,000 - $74,999  $75,000 - $99,999  8. $100,000 and above |
| Current Employment | Derived as a composite of the following two questions:   1. Are you working now, looking for work, retired, keeping house, a student, or something else? 2. Are you considered an essential worker? An essential worker is someone who was required to go to work even when stay at home orders were in place. |
| Housing Status | Derived as a composite of the following two questions:   1. What best describes the people at your home? 2. Are you currently living in transitional housing, staying in a shelter, or experiencing homelessness? |
| No. of Economic Challenges | Derived as the total number of economic challenges met from the following list:  In the past 6 months, have you or your family experienced the following challenge?   1. Getting the healthcare I need (including for mental health)? 2. Having a place to stay/live? 3. Getting enough food to eat? 4. Having clean water to drink? 5. Getting the medicine I need? 6. Getting where I need to go? |
|  |  |
| Health Risk Characteristics |  |
| Uninsured | What is the primary kind of health insurance or health care plan that you have now? Response options include: I do not have health insurance; Private (purchased directly through employment); Public (Medicare, Medicaid, Tricare) |
| Fair-Poor Health | Would you say your health in general is excellent, very good, good, fair, or poor? |
| Disability | Do you have a disability that interferes with your ability to carry out daily activities? Examples of daily activities include walking, climbing stairs, shopping, balancing a checkbook, bathing or dressing. |
| Any Drug Use | Derived as a composite of the following two questions:   1. Does the subject have a history of alcohol or substance use disorder for the study? 2. Does the subject have a history of intravenous drug use for the study? |
| Any Heavy Alcohol Use | How often do you have a drink containing alcohol? Heavy alcohol use is considered as consuming alcohol 4 or more times per week. |
| Any Mental Health Risk | Derived as a composite of the following two questions:   1. Does the subject have a history of depression? 2. Does the subject have a history of other mental health disorders? |
| No. of CVD Risk Factors | Derived as the total number of the following conditions met:   1. Do you now smoke cigarettes? 2. Overweight (derived from height and weight) 3. Do you have a history of diabetes? 4. Do you have a history of hypertension? 5. Do you have a history of cardiovascular disease? |
| No. of Chronic Condition Risk Factors | Derived as the total number of the following conditions met:  Does the subject have a history of:   1. Immunocompromised condition 2. Autoimmune disorder 3. Chronic kidney disease 4. Cancer 5. Asthma 6. COPD 7. Chronic lung disease 8. Sickle cell anemia 9. Other chronic condition |
| Access to COVID-19 Testing | Derived as a composite of the following two likert scale questions:   1. It is easy to get tested for COVID-19 2. I know where I can get COVID-19 testing in my community |
| Ever Received a Flu Vaccine | Have you ever received a flu vaccination? |
