# Supplementary material for: COVID-19 prevention is shaped by polysocial risk: A cross-sectional study of vaccination and testing disparities in underserved populations
Source: PLoS One. 2025 Jul 17;20(7):e0328779. doi: 10.1371/journal.pone.0328779 (PMC12270183; doi:10.1371/journal.pone.0328779)
Supplement: S1 Appendix — This appendix outlines the exclusion criteria for the study population, including project-level and participant-level exclusions based on missing data and other criteria. It provides detailed information on the reasons for excluding certain projects and participants from the study. (DOCX) [file pone.0328779.s003.docx]

**Supplemental Appendix 1. Exclusion Criteria**

Starting population: N = 453,048

*Project-level*

- Missing ≥80% on either primary outcome
  - 21 projects excluded (N=398,535 participants)
- Missing ≥80% on sex assigned at birth
  - 3 projects excluded (N=24,287 participants)
- Missing ≥80% on race or ethnicity
  - 0 projects excluded
- Projects with fewer than 20% of its participants with complete data across all independent variables of interest
  - 8 projects excluded (N=5,456 participants)
- Projects with fewer than 100 participants
  - 4 projects excluded (N=193 participants)

*Participant-level*

- Participants with missing data on BOTH primary endpoints
  - N=6,450 participants excluded
- Participants missing 100% of the data on clusters of independent variables of interest
  - N=309 participants excluded)

Additional project exclusions (using a “best judgment criteria”):

- Project 6: Missing rate of >55% across a cluster of “COVID-specific” independent variables.
- Project 7: Missing rate is ~40% or higher across all independent variables considered.
- Project 9: Missing rate is 70% or higher on all medical history independent variables and >40% on a “substance use” cluster of independent variables.
- Project 15: Missing 50%-70% on 12 of 13 “social risk” cluster of independent variables as well as 60% on a “substance use” cluster of independent variables.
- Project 30: Missing 100% on 3 of 5 “substance use” independent variables.
- Project 32: Missing >30% on half of the “COVID-specific” independent variables plus several others.
- Project 41: Has <100 participants and is missing 100% on 3 of 5 “substance use” independent variables.
- Project 70: Missing 100% on 6 of 13 “social risk” independent variables.
- Project 72: Missing 55% or higher on every single independent variable considered.
- Project S06: Missing >60% on 12 of 13 “social risk” independent variables and all “substance use” and “health status” independent variables.
- Project S07: Missing rate is 33% or higher for nearly all “substance use” and “health status” independent variables.
- Project S14: Missing >75% on all “substance use” independent variables and 16 of 17 “health status” independent variables.
- Project YMCF: Missing 100% of a cluster of “Economic Challenge” independent variables (would have to impute entire project for multiple variables.
