# Supplementary material for: COVID-19 prevention is shaped by polysocial risk: A cross-sectional study of vaccination and testing disparities in underserved populations
Source: PLoS One. 2025 Jul 17;20(7):e0328779. doi: 10.1371/journal.pone.0328779 (PMC12270183; doi:10.1371/journal.pone.0328779)
Supplement: Attachment — Submitted filename: POne Response to Review_Polysocial_Risk.docx [file pone.0328779.s006.docx]

**Dear Editors and Reviewers,**

We are grateful for the opportunity to resubmit our manuscript titled **COVID-19 Prevention Is Shaped by Polysocial Risk: A Cross-Sectional Study of Vaccination and Testing Disparities in Underserved Populations** to PLOS ONE

We appreciate the time invested by the PLOS ONE editors and the reviewers in providing meaningful insight for the direction of this manuscript. Following your suggestions, we have made the following major changes:

- **Clarified data collection dates** in the Methods section and the abstract.
- **Clarified that the test results were those provided by self-report prior to being tested for this study.** We deleted reference to data on test results obtained through testing after the survey. We ultimately decided not to include analysis of the results of testing after the survey because the results had very low positivity overall.
- **Revised the discussion** to avoid implying causality and to better align with the study's cross-sectional design.
- **Expanded the Public Health and Policy Implications section** to include more points pertinent to our findings.
- **Addressed minor comments** regarding formatting and grammar.
- **We updated file names** to adhere to PLOS ONE's style requirements
- **Noted in the updated title page** that the funders had “no role in study design, data collection and analysis, decision to publish, or preparation of the manuscript."
- **Updated our data availability statement** noting that data is available by request from the RADx-UP data Hub.
- **Uploaded and adjusted figure file** with the Preflight Analysis and Conversion Engine (PACE)
- **Included captions for Supporting Information file** at the end of the manuscript, and updated in-text citations to match.

**Detailed Responses to Reviewers' Comments**

**Reviewer #1:**

**Methods:**

- **There is inconsistency in data collection dates in the Methods section and the abstract.**
  - **Methods**: Between February 3, 2020 and April 21, 2023.
  - **Abstract**: Between October 2020–June 2024.
- **“This study used data from the RADx-UP Core Analytic Datasets version 1.6 collected by accessed through the CDCC on April 30, 2023 [40,43]”.**
  - This sentence is grammatically incorrect. The date is again inconsistent; this version says April 30, 2023, while the authors previously noted April 21, 2023, in their Methods.

**Author’s Response**: We appreciate the reviewer’s attention to these details. We have revised abstract and methods to clarify that data was collected by individual projects from February 2020 through April 21, 2023, and that the compiled data was accessed through the CDCC on April 30, 2023.

**Inclusion criteria:**

- The authors note the following inclusion criteria: “Participants were adults aged 18 years and older who underwent COVID-19 testing and were enrolled in a RADx-UP project”
- Later, they define one of their outcomes as COVID-19 testing.
- Were participants who never tested for COVID-19 included in the study?

**Author’s Response:** We clarified in the measures section that participants were surveyed prior to being tested for the study. In the survey, they were asked whether they had ever been tested for Covid-19. The outcome of never tested reflects their response to the survey prior to being tested for the study.

**In the measures section, we clarified that the testing outcome reflected the response to a survey question regarding their previous Covid-19 test prior to the survey. It is not the test result received from the Covid-19 test completed as part of the RADs-UP study. .**

“COVID-19 testing engagement was assessed through participants' history of testing (“Have you ever been tested for COVID-19?”) prior to their enrollment in the RADx-UP study. Prior test positivity rates were also examined among participants who had previously engaged in testing.”

**Analysis**:

- Can the authors report the extent of heterogeneity in individual projects included in the study for different outcome analyses? The authors have included Project as a clustering group in their analyses, but it may help if they can report how much heterogeneity was present as well. Just a suggestion though, the analysis in itself is adequate as well.

**Author’s Response:** Thank you for this suggestion. To this point, we have added this text to the statistical analysis section:

“Among the 14 projects included in the analysis, sample sizes ranged from 95 to 3,200 participants. The percentage of participants who met the primary vaccination outcome and the primary testing outcome ranged from 26.3% to 93.7% across projects and 46.5% to 98.9%, respectively. Within-project correlation was estimated to be 0.04 and 0.02 for the primary vaccine and testing outcomes, respectively, indicating very low correlation among subject measurements from the same project.”

**Results**:

• “These characteristics underscore the study's focus on underserved populations disproportionately impacted by the COVID-19 pandemic.”

- I believe this sentence would be more appropriate in Discussion rather than Results.

**Author’s Response:** We removed this statement.

**Discussion**:

• “This stark difference highlights how race, age, and region compound to create barriers to prevention.”

• Please note that this was a cross-sectional analysis of a dataset, which included multiple projects (with very different study designs). Since it did not entirely rely on prospective or randomized designs, it is not appropriate to establish causal inferences. The above sentence implies a cause-and-effect relationship. An alternative way to phrase this would be: “This stark difference highlights how the compound effect of race, age, and region may potentially create barriers to prevention.”
Alternatively, you can use phrases such as “associated with”, which do not imply causality.

**Author’s Response:** We appreciate this feedback and have adopted the reviewer’s suggestion.

• Same feedback for further causal statements:

**Author’s Response:** We appreciate this feedback and made appropriate edits to highlight association rather than causation

“This gap highlights the association between economic precarity and preventive health behaviors.”

“This suggests a strong association between established patterns of healthcare engagement and COVID-19 prevention behaviors.”

- The authors observed the highest disparities in vaccinations when evaluating health-related risk factors, but they also found that testing rates were similar in these groups. The provide the following interpretation:
- “This likely reflects the success of widespread testing availability through community sites and at-home options [45].”
- However, one may argue that vaccination was also available widespread similar to testing methods. All the other risk groups that the authors studied had consistent disparities in both vaccination and testing practices. It is interesting that the group with highest disparity in vaccination had no differences in testing practices**.** The authors should provide potential explanations for this.

**Author’s Response:** We realize that our initial statement was a little unclear. We clarified that the testing gap was narrower across all groups than the vaccination gap. While it was most pronounced in the health risk groups, it was also seen in other risk groupings. Our updated language includes:

“Notably, testing disparities were narrower than vaccination disparities across risk groups. This likely reflects the success of widespread testing availability through community sites and at-home options [45] and may also reflect the generally higher rates of testing compared to vaccination in our sample.”

• Social patterns in test results: Do these patterns reveal individuals with “truly” higher odds of testing positive, or do they simply reflect that these groups were tested more frequently? The patterns of people testing more often and people testing positive are concordant in the authors’ analyses, except for individuals in the Northeast region. These tested more frequently but did not test positive more frequently. What could be a potential explanation for this?

**Author’s Response:** This is a limitation and is now noted in the limitations sections. Of note, the northeast had the highest rates of having been testing and lowest positivity rates. One might suspect they were tested more frequently in the northeast. But we didn’t capture that information.

**Limitations**:

• Self-reported data also introduces concerns related to social desirability bias.
The authors could only study if the participants had ever been tested or not. They could not study the frequency of COVID-19 testing, which may influence the test positivity rates. Similarly, the authors studied whether the participants had ever received a COVID-19 vaccine, they did not study whether participants received all required vaccination doses (the complete series) or not.

**Author’s Response:** These points have been added to the updated limitations section

“While this study provides new insights into social risk stratification, several limitations must be acknowledged. **Its cross-sectional design limits causal inference, and reliance on self-reported data introduces potential recall and social desirability bias. Data did not include the frequency of COVID-19 testing, which may influence the positivity rates, or the completion of all recommended vaccine doses.”**

**Reviewer #2:**

***Conclusion***

• A significant chunk of 'conclusion' can be moved to 'Public Health and Policy Implications' section.

***Author’s Response:*** We have moved relevant parts of the conclusion to the Public Health and Policy Implications section and trimmed the conclusion accordingly.

***Public Health and Policy Implications***

• Authors need to update the Public health and policy implications section in discussion to include more points pertinent to their finding in this study, rather than a general importance of SDOH and Healthcare disparities.

**Author’s Response:** We have expanded this section to include more specific points related to our findings, such as the significant disparities in COVID-19 vaccination and testing among different polysocial risk profiles. The following was added to this section:

“Our findings highlight significant disparities in COVID-19 vaccination and testing among different polysocial risk profiles. For example, individuals experiencing intersecting high geo-demographic (Non-Hispanic Black, age 45, Southern residence), economic (low education, unemployment, financial hardship), or health-related risk factors (substance use, low CVD risk, no flu vaccination) were each significantly less likely to be vaccinated compared to groups with low geo-demographic, economic, or health risk profiles.

The utility of polysocial risk modeling as a predictive tool for identifying populations at highest risk of disengagement from preventive care can inform targeted precision public health interventions.

Beyond COVID-19, the polysocial risk framework has broader applicability for understanding disparities in chronic disease prevention, cancer screening, maternal and child health, and health-related social needs (HRSN) interventions.”

**Methods**

• Details of groupings mentioned in the last paragraph of methods should be described in the methods.

**Author’s Response**: We have edited this paragraph for clarity.

“To evaluate differences in preventive behavior adoption, we used our estimated models to predict the probability of vaccination and testing across representative population groupings. Three risk groups were considered: 1) geo-demographic risk groups, consisting of age, race, and geographic region of residence; 2) economic risk groups, consisting of education level, employment status, and number of economic challenges present (i.e., ability to get access to food, water, transportation, housing, needed medications, health care); 3) health risk groups, consisting of drug use (i.e., intravenous or any substance abuse), cardiovascular disease risk factors (i.e., smoking, vaping, overweight, diabetes, hypertension, and history of cardiovascular disease), and flu vaccination status. For each grouping, we compared populations with higher versus lower likelihood of engaging in these preventive measures (vaccination and testing). These groupings were selected based on commonly occurring combinations of characteristics in our dataset and were chosen to illustrate key differences in preventive measure engagement across demographic, economic, and health-related factors.”

**Editorial guidance:**

**Figures**

• While revising your submission, please upload your figure files to the Preflight Analysis and Conversion Engine (PACE) digital diagnostic tool, [https://pacev2.apexcovantage.com/](https://urldefense.com/v3/__https:/track.editorialmanager.com/CL0/https:*2F*2Fpacev2.apexcovantage.com*2F/1/010f0195e19a805d-7d9b794d-a32a-48af-9bc4-6c3fec2f5c15-000000/vA3_2EicaeQeijxwd3xunx9Jv85K3p5MOYpMKAysqPo=204__;JSUl!!FjuHKAHQs5udqho!JgJJpn5ig3VCZazgyMxVoXmeuxNoSv6DBuKONkaOKBqznE-RbLVunLHbQeS6a610C54E1O7LLFIkRRu_$). PACE helps ensure that figures meet PLOS requirements. To use PACE, you must first register as a user. Registration is free. Then, login and navigate to the UPLOAD tab, where you will find detailed instructions on how to use the tool. If you encounter any issues or have any questions when using PACE, please email PLOS at [figures@plos.org](mailto:figures@plos.org). Please note that Supporting Information files do not need this step.

**Author’s Response:** We have completed this step and uploaded the adjusted files. PACE made these adjustments:

- Resolution is changed to 300 PPI
- TIF file is converted to a valid TIF file.

**Supporting information**

• Please include captions for your Supporting Information files at the end of your manuscript, and update any in-text citations to match accordingly. Please see our Supporting Information guidelines for more information: http://journals.plos.org/plosone/s/supporting-information.

**Author’s Response:** We have included Supporting Information Captions at the end of the manuscript as follows:

**“S1 Supporting Information**: This file includes the following:

**S1 Table. Study Project Metadata.** This table provides detailed metadata for various study projects, including geographic location, primary and secondary populations, study design, study setting, mode of data collection, and vaccine availability p

hase. It also includes exclusion criteria for the study population and descriptions of prevention behaviors and population characteristics

**S2 Table**. **Description of Prevention Behaviors and Population Characteristics.** This table describes prevention behaviors and population characteristics, including survey questions and derivation details for primary outcomes such as COVID-19 vaccination, testing, and positive test results. It also covers demographics, economic risk characteristics, health risk characteristics, and access to COVID-19 testing

**S1 Appendix. Exclusion Criteria.** This appendix outlines the exclusion criteria for the study population, including project-level and participant-level exclusions based on missing data and other criteria. It provides detailed information on the reasons for excluding certain projects and participants from the study”

**We believe that these revisions have significantly improved the manuscript and addressed the reviewers' concerns. We hope that the revised manuscript meets the standards for publication in PLOS ONE.**

**Thank you for your consideration.**
